# Modulation of the Dipole Potential of Model Lipid Membranes with Phytochemicals: Molecular Mechanisms, Structure–Activity Relationships, and Implications in Reconstituted Ion Channels

**DOI:** 10.3390/membranes13040453

**Published:** 2023-04-21

**Authors:** Svetlana S. Efimova, Olga S. Ostroumova

**Affiliations:** Laboratory of Membrane and Ion Channel Modeling, Institute of Cytology of Russian Academy of Science, Tikhoretsky Ave. 4, 194064 St. Petersburg, Russia

**Keywords:** phytochemicals, flavonoids, alkaloids, saponins, lipid bilayers, membrane dipole potential, ion channels, anti-microbial agents

## Abstract

Phytochemicals, such as flavonoids, stilbenoids, alkaloids, terpenoids, and related compounds, have a wide range of useful pharmacological properties which cannot be ascribed to binding to a single peptide or protein target alone. Due to the relatively high lipophilicity of phytochemicals, the lipid membrane is thought to mediate their effects via changes in the properties of the lipid matrix, in particular, by modulating the transmembrane distribution of the electrical potential and, consequently, the formation and functioning of the ion channels reconstituted in the lipid bilayers. Therefore, biophysical studies on the interactions between plant metabolites and model lipid membranes are still of interest. This review represents an attempt to provide a critical analysis of a variety of studies on altering membranes and ion channels with phytochemicals via disturbing the potential drop at the membrane–aqueous solution interface. Critical structural motifs and functioning groups in the molecules of plant polyphenols (alkaloids and saponins are identified) and the possible mechanisms of dipole potential modulation with phytochemicals are discussed.

## 1. Introduction

The functional classification of secondary metabolites is used in conjunction with other classifications since the compounds of different structures might be included in one group according to their functions. Using the chemical classification, secondary metabolites are divided into three main classes: polyphenols (see below in Section 1.1), alkaloids (see below in Section 1.2), and isoprenoids (see below in Section 1.3).

### 1.1. Polyphenols

**Figure 1:**
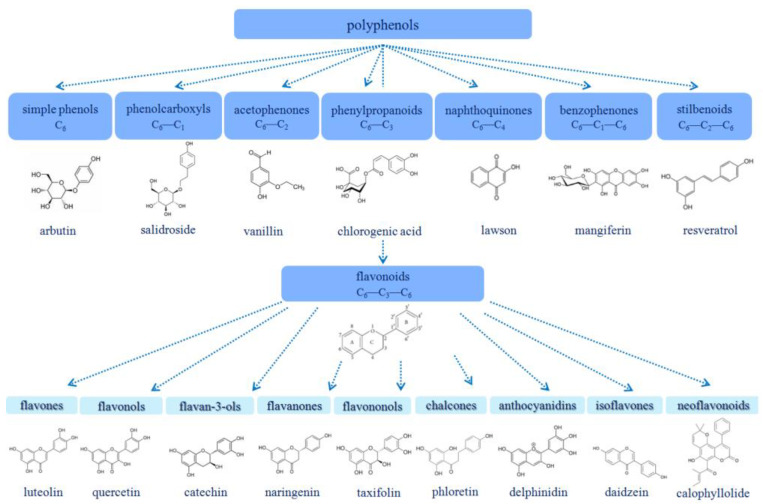
The classification of plant polyphenols and the chemical structures of some representatives.

Phenolic compounds are aromatic molecules containing one or more hydroxyl group in the benzene rings [1]. Substances with one or more than two hydroxyl groups are called phenols and polyphenols, respectively. Preclinical and clinical studies support the protective action of polyphenols in cardiovascular diseases [2], neurodegenerative diseases [3], and cancer [4] due to high anti-oxidant activity, which is related to the number of OH groups. Phenolic compounds are mainly represented in plants by flavonoids, lignans (polymer phenolic compounds), and tannins, and are accumulated in flower petals, fruits, roots (licorice), shoots (motherwort), etc. About 8000 plant phenolic compounds are known to date, and their number is growing every day. The classification of phenolic compounds occurs depending on the number of aromatic rings and the number of carbon atoms attached to the ring. Figure 1 demonstrates the classification of plant polyphenols with typical chemical structures in each class: simple phenols, without extra carbon atoms; phenolcarboxyls, containing one additional carbon atom; acetophenones, containing two additional carbon atoms; phenylpropanoids, containing three additional carbon atoms; naphthoquinones, containing four additional carbon atoms; benzophenones, containing two aromatic rings linked by a bridge of one carbon atom; stilbenoids, containing two aromatic rings linked by two carbon atoms; and flavonoids, containing three aromatic rings linked by three carbon atoms.

Flavonoids are the most numerous groups of phenolic compounds [5,6,7]. There are at least 11 subclasses of flavonoids. The most studied subclasses are shown in Figure 1; they include flavones, flavonols, flavan-3-ols, flavanones, flavononols, chalcones, dihydrochalcones, anthocyanidins, isoflavonoids, and neoflavonoids. All flavonoid molecules contain two benzene rings, which are usually connected by a heterocycle with one oxygen atom or propane fragment and a carboxyl group (Figure 1). The aromatic rings of flavonoids might contain a number of hydroxyl substituents, and some of them even contain one more phenyl substituent.

Stilbenoids have a similar structure with two aromatic rings and several hydroxyl groups [8]. However, unlike flavonoids, they have a diene chain like an ‘elastic stick’ connecting the two aromatic rings instead of the third ring structure found in flavonoids or the oxidized propane chain in chalcones. The stilbenoid resveratrol is involved in the modulation of different signaling pathways in the cell [9] and provides anti-oxidant [10], anti-inflammatory [11], anti-microbial [12], anti-neoplastic [13], anti-diabetic [14] and cardio- and neuroprotective activities [15,16]. The description of each subclass of flavonoids and the most studied representatives are given below.

Flavones have a double bond between C_2_ and C_3_ in the flavonoid skeleton; the molecules are not substituted at the C_3_ position, and they are oxidized at the C_4_ position [17]. Flavones are components found in vegetables, fruits, nuts, seeds, and tea. They have been reported to possess anti-malarial, anti-microbial, anti-tuberculous, anti-allergic, anti-oxidant, anti-inflammatory, and anti-cancer activities [18,19,20,21,22]. The main representative of this subclass is luteolin, which is found in carrots, celery, olive oil, mint, and chamomile; it exhibits anti-inflammatory effects and improves mental performance [23]. Moreover, luteolin showed a binding affinity for the ACE-2 receptor in silico [24]. The flavone baicalein and its glucuronated derivative baicalin, produced by *Scutellaria baicalensis*, are used for the treatment of various types of cancer, hepatitis, T-cell leukemia, fever, inflammation, and several kinds of infections [25,26]. Apigenin was found to be a potent inhibitor of cell proliferation and angiogenesis in the human endothelial cells [27].

Flavonols not only contain a carbonyl group at the C_4_ position, but they also have a hydroxyl group at the C_3_ position of the pyran C-ring. Grape skin flavonols are found in the form of glycosides, quercitrin, isoquercitrin, quercetin-3-monoglucoside, quercetin-3-monoglucuronoside, and myricetin-3-monoglucoside, and, in wine, they are found in the form of aglycones, kaempferol, quercetin, and myricetin [28]. Kaempferol exhibits dramatic anti-inflammatory properties and has been used to cure many acute and chronic inflammation-induced diseases, such as intervertebral disc degeneration, colitis, post-menopausal bone loss, and acute lung injury [29]. Quercetin and myricetin demonstrate wide biological activities, including anti-oxidant, anti-inflammatory, anti-bacterial, anti-viral, gastroprotective, and immune-modulatory actions [30,31,32]. It has been recently demonstrated that quercetin has anti-viral activity, against coronavirus in particular, and a binding affinity for the ACE-2 receptor in silico [24,33,34].

Flavan-3-ols (also called catechins) were named after *Acacia catechu* because they were first isolated from its wood. Their molecules contain a hydroxyl group at the C_3_ position and are not oxidized at the C_4_ position. Catechins are widely distributed in plants, demonstrate P-vitamin activity, and are used in the treatment of diseases that are associated with capillary dysfunction or edema of a vascular origin [35]. It is widely recognized that green tea containing catechins, (-)-epicatechin-3-gallate, (-)-epigallocatechin, (-)-epicatechin, and (-)-epigallocatechin-3-gallate, protects against cardiovascular diseases, stimulates weight loss, and has protective effects against neurodegeneration, and against Alzheimer’s and Parkinson’s disease in particular [35].

Flavanones are characterized by a pale-yellow color; their structures contain a carbonyl group in the C_4_ position and do not have a double bond in the C-ring. They are found in tomatoes and aromatic plants, such as mint, but their major sources are citrus fruits [36,37,38], especially grapefruits [39]. The flavanone naringenin has potential therapeutic activity in neurological, cardiovascular, gastrointestinal, and malignant disorders [40]. Liquiritigenin demonstrates high anti-inflammatory activity [41] and promising neuroprotective effects and, therefore, may be useful in developing a specific treatment for Alzheimer’s disease [42]. Rutin has P-vitamin activity and promotes the assimilation of ascorbic acid, and is used along with naringenin for the treatment of obesity [43].

Flavanonols have a structure similar to that of flavanones with an additional hydroxyl group in the C_3_ position. Most flavanonols were isolated from coniferous and hardwood wood species. Taxifolin and aromadendrin are the main representatives of this class. Taxifolin exhibits anti-oxidant, anti-toxic, regeneration, and anti-edematous activities [44,45].

Chalcones and dihydrochalcones are often considered to be open pyran C-ring flavonoids, and they demonstrate significant anti-oxidant, anti-inflammatory, anti-tumor, anti-diabetic, and anti-bacterial properties [46]. Cardamonin exhibits anti-inflammatory activity and, therefore, has the ability to prevent tumorigenesis [47]. Several studies have shown that the anti-proliferative effects of a substance on cancer cells are associated with its anti-oxidant properties [48]. The dihydrochalcone phloretin and its glycoside phlorizin are known as inhibitors of glucose transport in the intestinal and renal epithelium cells [49]. Phloretin is also able to inhibit the transport of urea in various cells, including the cells of the renal epithelium, liver, and erythrocytes [50].

Anthocyanidins are present in plants as glycosides. These plant pigments give color to flowers, fruits, and leaves. It is known that several anthocyanidins differ by the radicals in the 3′- and 5′-positions of the B-ring. The biological role of anthocyanins has not yet been fully established. There is information on the anti-inflammatory, anti-oxidant, and cancer-inhibitory properties of anthocyanidins [51,52].

Isoflavones possess a B-ring attached at the C_3_ position of the C-ring. Isoflavonoids have shown anti-oxidant effects due to their free-radical scavenging capacity by donating the hydrogen atoms of the hydroxyl group attached to the benzene ring, thus protecting against oxidative damage and macromolecule damage and reducing low-density lipoproteins [53]. Isoflavonoids have been found in two chemical forms; aglycones (biochanin A, daidzein, and genistein) and glycosides (daidzin and genistin). These isoflavonoids demonstrate vitamin activity and help to strengthen the bones [54]. The synthesis of isoflavonoids is characteristic of leguminous plants, where they act as phytoalexins.

Neoflavonoids are a class of polyphenolic compounds with a 4-phenylchromene backbone at the C_2_ position. Neoflavonoids display a variety of pharmacological activities, such as anti-osteoporosis, anti-inflammatory, anti-tumor, anti-androgen, anti-allergic, and anti-oxidative activities [55,56].

### 1.2. Alkaloids

**Figure 2:**
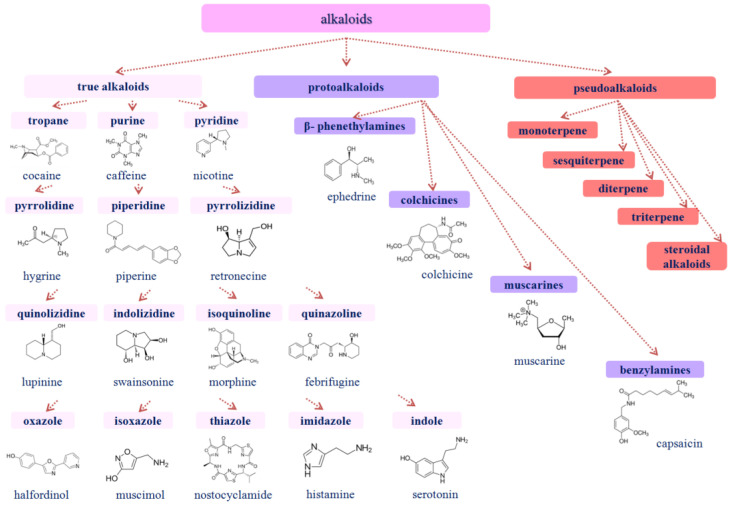
The classification of plant alkaloids and the chemical structures of some representatives.

Alkaloids are heterocyclic compounds containing one or more nitrogen atoms and are characterized by significant structural diversity. They are usually divided into several main groups [57]. As a rule, alkaloids are found in plants in the form of salts of malic, tartaric, citric, and other acids. They are divided into true alkaloids; protoalkaloids, which have nitrogen in the side chain; and pseudoalkaloids, which are synthesized through transamination [58] (Figure 2). Figure 2 demonstrates the typical chemical structures of alkaloids belonging to different chemical groups. Vasodilators, anti-hypertensive and anti-arrhythmic compounds, anesthetics, and analgesics have been found among plant alkaloids [59,60]. Alkaloids also demonstrate anti-proliferative, anti-bacterial, and anti-oxidant properties [61]. The therapeutic potential of alkaloids determines their wide industrial application. The immunomodulatory properties of plant alkaloids are also actively discussed [62]. Information on the pharmacological action of some representatives of alkaloids is presented below.

True alkaloids are obtained from amino acids; they share a nitrogen-containing heterocyclic ring and have potent biological activity. Almost all true alkaloids are bitter in taste and are solid, except nicotine, which is a brown bitter liquid [57]. Various amino acids, such as L-phenylalanine, L-tyrosine, L-ornithine, L-histidine, and L-lysine, are the main sources of true alkaloids [63,64]. The tropane derivative cocaine was used as a local numbing agent and vasoconstrictor to help control pain and bleeding during surgery [65]. The anti-malarial effect of quinine has been shown [66]. The piperidine derivative piperine and the purine derivative caffeine possess therapeutic potential for Alzheimer’s disease, Huntington’s disease, Parkinson’s disease, cerebral ischemia, and schizophrenia [67,68]. Moreover, it has been shown that the administration of oral curcumin with piperine as an adjuvant symptomatic therapy in COVID-19 treatment could substantially reduce morbidity and mortality [69].

Protoalkaloids contain a nitrogen atom which is derived from an amino acid but not part of the heterocyclic ring system. Colchicine and capsaicin are very popular examples of protoalkaloids. Colchicine demonstrates protective cardiovascular effects [70] and putative efficacy in the treatment of patients with COVID-19 [71]. Using various types of oncological models, the modulation of signaling pathways, oncogenes, and tumor suppressor genes through the addition of capsaicin has been shown [72], and the possibility of combined drugs based on alkaloids and other anti-cancer compounds is being discussed [73,74].

The basic carbon skeleton of pseudoalkaloids is not directly derived from amino acids; instead, they are synthesized through an amination or transamination reaction by the forerunners or postcursors of amino acids [63]. The pseudoalkaloid solanidine is responsible for neuromuscular syndromes via cholinesterase inhibition [75,76].

### 1.3. Isoprenoids

**Figure 3:**
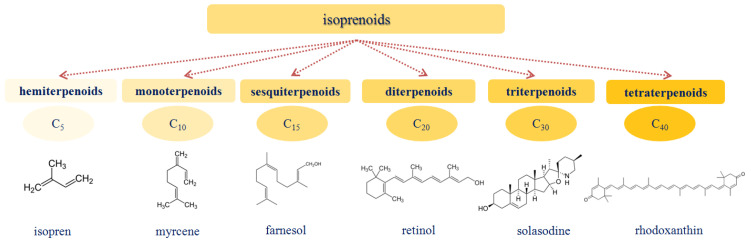
The classification of isoprenoids and the chemical structures of some representatives.

Isoprenoids (also called terpenoids) are compounds composed of several C_5_H_8_ isoprene units. The isoprene skeleton can be found in naturally occurring compounds, such as carotene, phytol, retinol (vitamin A), tocopherol (vitamin E), and squalene [77]. Figure 3 demonstrates the classification of isoprenoids and the chemical structures of their representatives.

Monoterpenes and sesquiterpenes include more than 3000 compounds and are highly volatile liquids with an odor [78]. They are the main components of essential oils [79]. Diterpenes are also a large group that contains several thousand structures, possesses a rich pharmacology, and includes important compounds such as retinol and phytol [80]. Diterpenes are widely represented in the resins of spruce, pine, fir, and other coniferous trees and are found in the form of cyclic structures. Triterpenes are the components of plants and accumulate in the form of acid esters and glycosides [81,82]. Triterpene glycosides (i.e., saponins) are surfactants and are poisonous to animals. The pronounced hemolytic activity of saponins is explained by their interaction with cholesterol in the erythrocyte membrane [83,84,85]. The great structural diversity of saponins that can be found in nature is due to the presence of various sugars, such as branching sugars, and sapogenins [86,87]. Tetraterpenes include one structural group called carotenoids. At the present time, about 500 representatives of this group are known, which are synthesized in plants and have a color ranging from yellow to red orange [88].

The classification of saponins is based on the structure of aglycones: triterpenoid saponins are synthesized directly from squalene and contain 30 carbon atoms. The structural diversity of saponins explains their diverse physicochemical, pharmacological, and biological properties, which determine their applied significance in the food, cosmetic, and pharmaceutical fields [89]. The pharmacological actions of saponins and related compounds include anti-inflammatory, anti-nociceptive, anti-pyretic, anti-allergic, and anti-cancer properties [90,91]. The steroid alkaloid solasodine exhibits diuretic, anti-cancer, anti-fungal, cardiotonic, anti-spermatogenetic, anti-androgenic, immunomodulatory, and anti-pyretic activities in the central nervous system [92].

## 2. Phytochemicals Alter the Electrical Properties of the Model Lipid Membranes

An analysis of the literature has shown that the diverse mechanisms of the biological actions of plant metabolites might be associated with the amphiphilicity of their molecules, which determines an ability to interact with cell membranes. Amphiphilic compounds, characterized by the presence of an electric charge and/or significant dipole moment, when interacting with biological membranes, can affect their electrical properties.

The electrical properties of the membrane primarily include the interfacial electrical potential jump called the membrane boundary potential, which consists of two components; the surface and the dipole potential. The first component is related to the charges of the membrane lipids and adsorbed molecules, the ionizing groups of the amino acid residues of the membrane-associated proteins, and the electrolyte counterions in the aqueous solution which, altogether, create a double electric layer. The theoretical consideration of this phenomenon was developed in the works of Gui and Chapman and subsequently supplemented by Stern [93].

Liberman and Topaly suggested the existence of one more potential jump at the membrane–aqueous solution interface in 1969 [94]. Hladky and Haydon first used the term “dipole potential” in 1973 and supposed that it is related to lipid dipoles [95]. At present, the dipole potential is considered to be the unshielded part of the potential jump at the interface which arises due to the specific orientation of the dipoles of the membrane lipids and adsorbed water molecules. As a result, the electric potential of the hydrocarbon core of the membrane turns out to be more positive relative to the aqueous phase surrounding the bilayer, which prevents the penetration of cations and facilitates the transmembrane transport of anions [96,97]. From the above definition of the dipole potential, it follows that its value can be changed in two fundamentally different ways, namely, by changing the hydration of the membrane or by incorporating compounds with high dipole moments oriented along the normal membrane surface.

The relationship between the value of the membrane dipole potential, the dipole moment of the molecules located at the interface, the surface density of the dipoles, and the dielectric constant of the membrane is expressed by the Helmholtz equation [96]:

(1) $$\varphi_{d}=\frac{\mu \cdot n}{\varepsilon_{0}\cdot \varepsilon },$$

where *μ* is the effective dipole moment projection to the normal membrane surface, *n* is the effective surface density of the molecular dipoles, *ε* is the dielectric permeability of the membrane, and *ε*_0_ is the permeability of the free space.

The value of the membrane dipole potential depends on its lipid composition and varies from 200 to 400 mV [97,98,99,100,101,102]. It has been shown that the membrane dipole potential affects the peptide–lipid interactions, in particular, the membrane fusion caused by the virus fusion peptides [103,104]; the binding of amphiphilic drugs with membranes, local anesthetics in particular [105,106,107]; and the functioning of different ion channels, especially those formed by anti-microbial peptides and lipopeptides [108,109,110,111,112,113,114,115,116,117].

**Figure 4:**
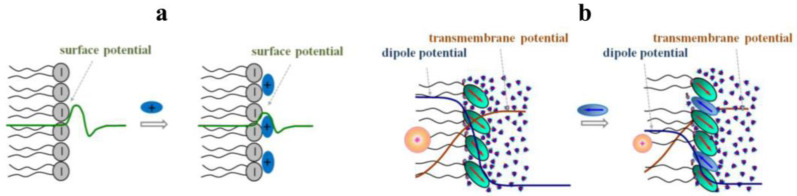
The schematic representations of the decrease in the absolute value of membrane surface (green curves) (**a**) and dipole (dark blue curves) potential (**b**) at addition of phytochemicals (blue ovals). The applied transmembrane voltage (brown curves) remains constant while dipole potential profile is altered due to the adsorption of the plant molecules. This results in dramatical changes in the inter-membrane electric potential, which is shown as virtual plus in the center of hydrocarbon core (orange cycle).

As has been noted above, small amphiphilic molecules and plant metabolites in particular can influence the surface and dipole components of the membrane boundary potential. This is schematically illustrated in Figure 4.

Using different ionophores and lipophilic ions, Andersen et al. [118] found that dihydrochalcone phloretin significantly increases the cationic conductance of the membranes and decreases the anionic conductance of the membranes. Similar results were obtained by Melnik and colleagues [119]. The authors suggested that the introduction of phloretin into the bilayer leads to a decrease in the membrane dipole potential. According to Bechinger and Seelig [120], this occurs due to the reorientation of the dipoles of the lipid molecules and the changes in the hydration of the bilayer. Cseh and Benz suggested that the adsorption of phloretin into the membrane is accompanied by changes in the lipid packing density [121]. A comparative study on the dipole-modifying effects of a number of plant polyphenols, including flavonoids, belonging to different structural groups was carried out by [117,122,123]. Table 1 presents the maximum reduction in the boundary (Δφ*_b_*(max)) and dipole (Δφ*_d_*(max)) potential of bilayers composed of pure phosphocholine. By comparing the Δφ*_b_*(max) and Δφ*_d_*(max) values, one can assume the presumable role of the dipole component (Table 1).

**Figure 5:**
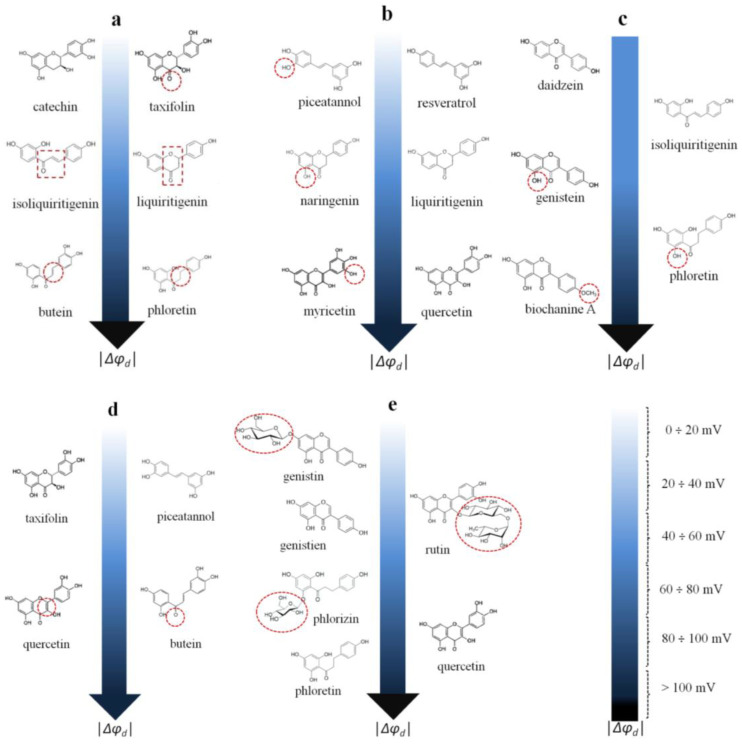
The relationships between the structure of polyphenols and their dipole-modifying ability. (**a**) The independence of dipole-modifying effect of polyphenols on the presence of the carbonyl group, heterocycle and double bond in the fragment linking the phenolic rings of molecules (from top to bottom); (**b**) The independence of dipole-modifying effect on inclusion of an additional OH group in the molecules of flavanones, flavonols, and stilbenoids (from top to bottom); (**c**) The dependence of dipole-modifying ability of isoflavones (left side) and chalcones/dihydrochalcones (right side) on the inclusion of additional OH- and methyl groups; (**d**) The dependence of dipole-modifying ability of flavonoid on the presence of the double bond in the heterocycle (left side) and the oxidation and length of the fragment linking the phenolic rings (right side); (**e**) The dependence of dipole-modifying ability of flavonoids on the glycosylation of isoflavones/dihydrochalcones (left side) and flavonols (right side).

By analyzing the data obtained with the polyphenols presented in Table 1, one can draw the following conclusions:

(1) The ability of chalcones to reduce the boundary/dipole potential increases in the following order: 4′-hydroxychalcone ≈ isoliquiritigenin (about −40 mV) ≤ cardamonin ≈ licochalcone A (about −60 ÷ −70 mV) < butein −120 mV). Despite the lower lipophilicity of butein among the other tested chalcones, its great efficiency might be explained by its higher dipole moment, which is probably related to the electron density shift in the A and B rings produced by the four hydroxyl groups.
(2) The chalcone butein and the dihydrochalcone phloretin are almost equally effective (the Δφ*_b_*(max) values coincide within the estimation error). This might indicate that the presence/absence of a double bond in the propane fragment linking the phenolic rings in the molecule of butein/phloretin, which significantly affects the mobility of the rings relative to each other, is not of key importance (Figure 5a).
(3) The chalcone isoliquiritigenin and the flavanone liquiritigenin are almost equally effective (Δφ*_b_*(max) values coincide within the measurement error), indicating that cyclization (the formation of a heterocycle) does not practically affect the ability of the compounds to modify the potential jump at the membrane–aqueous solution interface (Figure 5a).
(4) The exclusion of the carbonyl group from the structure (catechin compared to taxifolin, Δφ*_b_*(max) does not exceed 6 mV) does not affect the compound’s dipole-modifying effect (Figure 5a).
(5) The inclusion of an additional OH group in the molecules of flavanones (naringenin compared to liquiritigenin, Δφ*_b_*(max) ≈ −70 mV)), flavonols (myricetin compared to quercetin, Δφ*_b_*(max) ≈ −100 mV)), and stilbenoids (piceatannol compared to resveratrol, Δφ*_b_*(max) ≈ −10 mV) does not alter the dipole-modifying properties of the compounds (Figure 5b). This is not true in the case of chalcones/dihydrochalcones (phloretin (about −150 mV) compared to isoliquiritigenin (about −40 mV)), or isoflavones (genistein (about −70 mV) compared to daidzein (about −20 mV)) (Figure 5c).
(6) The methylation of the hydroxyl group in the B-ring of biochanin A compared to genistein leads to a significant potentiation of the dipole-modifying ability of isoflavones (Figure 5c).
(7) The reduction of the double bond in the heterocycle eliminates the dipole-modifying ability of the compound (taxifolin (about 0 mV) compared to quercetin (about −100 mV)) (Figure 5d). This effect can be explained by the difference in the dipole moments of the structurally related flavononols and flavonols.
(8) The replacement of the oxidized propane chain connecting the two aromatic rings in the chalcone butein with the diene chain in the stilbenoid piceatannol eliminates the dipole-modifying properties (Figure 5d).
(9) All glycosides are less effective at modulating the boundary potential than the related aglycones (phlorizin (about −90 mV) vs. phloretin (about −150 mV); rutin (about −40 mV) vs. quercetin (about −100 mV); and genistin (about −10 mV) vs. genistein (about −70 mV)) (Figure 5e).

**Figure 6:**
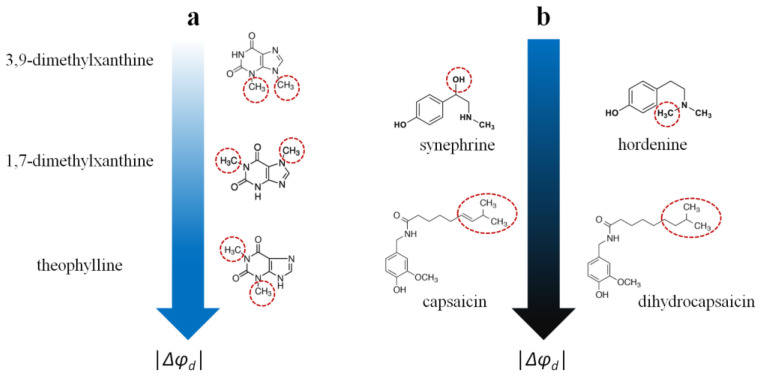
The relationships between the structure of alkaloids and their dipole-modifying ability. (**a**) The dependence of dipole-modifying ability of xanthines on the localization of the methyl groups; (**b**) The independence of dipole-modifying effect of protoalkaloids on the presence of OH-group and the double bond in the side chain of phenylethylamines and benzylamines respectively (from top to bottom). The color intensity designation is shown in the caption of Figure 5.

Table 1 also presents the maximum magnitude of reduction in the boundary and dipole potential of bilayers composed of pure phosphatidylcholine in the presence of different alkaloids according to [125]. The high structural diversity of the presented series of alkaloids allows us to draw only a few structure–function parallels, while a more detailed penetration requires the systematic testing of many structurally similar analogues in each subgroup. By comparing the structures of the tested alkaloids and their dipole-modifying ability (Table 1), one notices the following:

(1) The xanthine derivatives caffeine, pentoxifylline, 3,9-dimethylxanthine, and 7-(β-hydroxyethyl)theophylline do not affect the potential jump at the membrane–aqueous solution interface. The ability of the other tested xanthines to reduce the boundary/dipole potential increases in the following series: 1,7-dimethylxanthine ≈ 3-isobutyl-1-methylxanthine (about −20 mV) ≤ theophylline (about −40 mV). It can be assumed that the orientation of the dipole moment of xanthines relative to the normal membrane surface, which strongly depends on the type and localization of the hydrophobic substituents, is of decisive importance (Figure 6a).
(2) The pronounced ability of the benzylamines capsaicin and dihydrocapsaicin to influence the membrane boundary/dipole potential (about −120 mV) can be associated with their high lipophilicity and polarity. Moreover, the saturation of the side chain (dihydrocapsaicin compared to capsaicin) is irrelevant for the dipole-modifying properties of benzylamines (Figure 6b).
(3) The derivatives of β-phenylethylamine, synephrine and hordenine are almost equally effective (Δφ*_b_*(max) values coincide within the measurement error), indicating that the presence of an additional OH group in the side chain (synephrine compared to hordenine) does not affect the compounds’ dipole-modifying effect (−30 ÷ −40 mV) (Figure 6b).
(4) One can also note a significant decrease in the boundary/dipole potential in the presence of quinine, piperine, melatonin, colchicine, and conessine. The absence of information on several structurally similar compounds does not allow one to draw any strictly defined conclusions, and only some trends can be noted. The significant dipole-modifying activity of the effects of quinine and melatonin (about −30 mV) might be related to their structurally close quinoline and indole fragments. The ability of piperine to reduce the boundary/dipole potential (about −50 mV) might be associated with its piperidine fragment and is unlikely to be related to the piperonyl moiety, which is also present in the structure of inactive berberine. The attachment of dimethylamine to the A ring of the steroid core in the molecule of conessine instead of the hydroxyl group in the molecules of solasodine and solanidine might be responsible for the slight dipole-modifying effect of the first molecule (about −20 mV).

**Figure 7:**
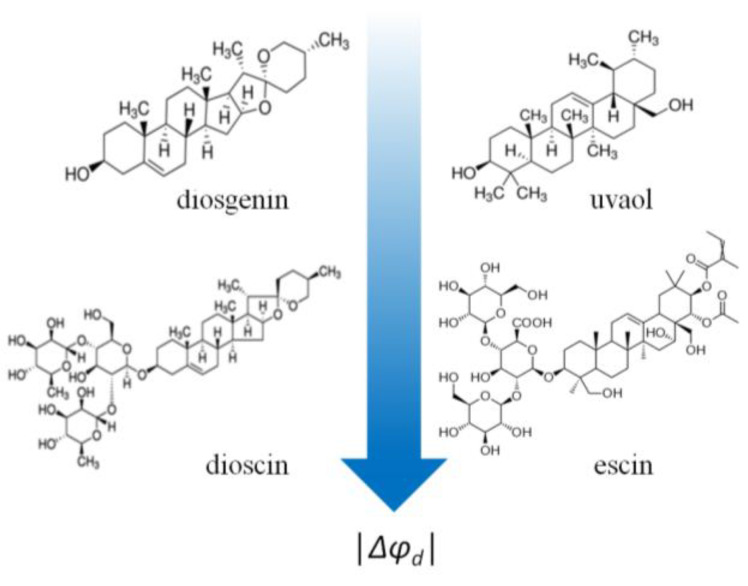
The relationships between the structure of saponins and related compounds and their modifying ability. Glycosylated analogs are more effective in modulating the membrane boundary potential than the corresponding aglycones independently of sapogenin core structure, steroid (left side) or triterpenoid (right side). The color intensity designation is shown in the caption of Figure 5.

By comparing the structures of all the tested saponins and related compounds to their potential-modifying properties (Table 1), one notices the following:

(1) Contrary to polyphenols, all glycosylated analogs (saponins: digitonin, tribulosin, dioscin, and escin) are more effective in modulating the membrane boundary potential (Δφ*_b_*(max) = −20 ÷ −50 mV)) than the corresponding aglycones (sapogenins: diosgenin, uvaol, lupeol, betulin, solasodine, and solanidine) (Δφ*_b_*(max) does not exceed −6 mV) (Figure 7).
(2) Dipole-modifying effects do not depend on the structure of the sapogenin (steroid or triterpenoid). Steroids (diosgenin, solasodine, and solanidine) and triterpenoids (uvaol, lupeol, and betulin) are all ineffective (Figure 7).

**Figure 8:**
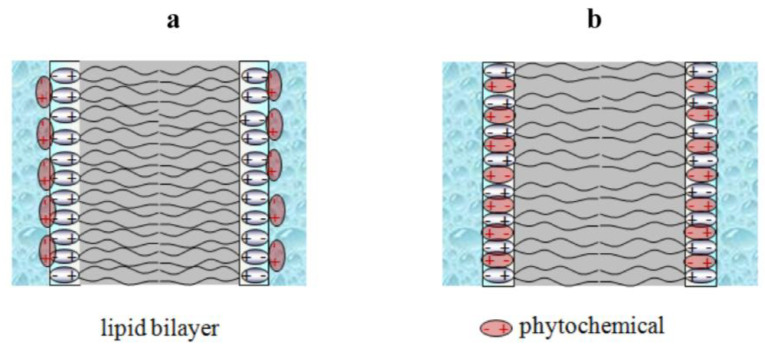
Two different ways of modulating dipole potential with various phytochemicals: (**a**) change in the membrane hydration and (**b**) intercalation of a compound’s own dipoles into the membrane along with disordering of membrane lipids. The grey color indicates the hydrocarbon core of the lipid bilayer; the blue color indicates the membrane bathing solution.

The different effect of glycosylation on the dipole-modifying ability of flavonoids and sapogenins indicates fundamental differences in the mechanisms of the modulation of the electric potential jump at the bilayer–aqueous solution interface by these compounds. Taking into account the fact that only glycosylated sapogenins, i.e., true saponins, which cannot significantly immerse into the bilayer, are able to reduce the bilayer boundary potential, it can be thought that they affect the potential jump by changing membrane hydration (Figure 8a). In confirmation, digitonin, tribulosin, dioscin, and escin do not affect the φ*_b_* of membranes composed of hexadecyl oleoyl phosphatidylcholine [126]. It is believed that the carbonyl in the ester group linking the hydrocarbon chain to the glycerol fragment of phosphatidylcholine mainly determines the number of water molecules that bind to the membrane [125,127]. Hexadecyl oleoyl phosphatidylcholine has one ether and one ester group instead of the two ester links in the molecules of the phosphatidylcholines mentioned in Table 1, which defines the different structure of the hydration layers of membranes composed of ester and ether phosphatidylcholines and, consequently, the possibility of it being restructured by saponins.

Unlike saponins, some chalcones/dihydrochalcones, piperine, and benzylamines, whose molecules have relatively high octanol–water distribution coefficients (Table 1), are able to incorporate into the membrane and affect its dipole potential (Figure 8b). The latter may be due to the relatively high dipole moments of their molecules, which can be oriented opposite to the existing total dipole moment of the membrane-forming lipids and water sorbed on the surface of the bilayer, and may be due to a decrease in the packing density of the lipids (i.e., a decrease in the surface density of the dipoles, *n*). The latter assumption is confirmed by a decrease in the melting temperature of saturated phosphocholines of more than 1 °C upon the incorporation of phloretin, 4-hydroxychalcone, butein, cardamonin, isoliquiritigenin, naringenin, licochalcone A, biochanin A, piperine, capsaicin, and dihydrocapsaicin [117,125,128]. The exceptions are highly hydroxylated flavonols, such as quercetin and myricetin, which significantly reduce the dipole potential of the membrane (Table 1) but do not practically affect the packing density of the lipids in the membrane [129]. This indicates that the mechanism of the change in the interfacial electric potential jump during their adsorption into the bilayer is similar to that of saponins (an alteration in membrane hydration) (Figure 8a).

## 3. The Role of Phytochemicals in the Formation and Functioning of the Ion Channels Formed by Anti-Microbial Agents

The study of the formation and functioning of the ion channels formed by anti-microbial agents is one of the central problems of modern molecular biology and pharmacology due to the need to overcome the antibiotic resistance of pathogenic strains. In this case, the small-molecule-induced alteration in the lipid matrix is of key importance because it might enhance the pore-forming activity of the anti-microbial agent.

It is well known that the membrane dipole potential affects the pore-forming activity of anti-microbial peptides and lipopeptides, such as gramicidin A (GrA) [110,125,126,130,131], alamethicin [108,111,132], cecropins (CeC) [116,125], syringomycin E (SrE) [112,117,125], surfactin (SuF) [115], polymyxin B (PmB) [133], the lantibiotic nisin (NiS) [134], and the polyene macrolide antibiotic amphotericin B (AmB), at their symmetrical addition [135].

Table 2 summarizes the data concerning the changes in the properties of single ion channels when modulating the membrane dipole potential with different phytochemicals. The dimers of the *Bacillus brevis* peptide GrA in planar lipid bilayers form symmetrical pores with practically ideal cation selectivity [130,136,137,138]. The one-sided addition of the anti-fungal lipopeptide *Pseudomonas syringae* SrE causes the appearance of asymmetric lipopeptide-lipid pores of a conical shape with predominant anion selectivity [139,140]. The two-sided addition of AmB, the anti-fungal polyene macrolide antibiotic from the *Streptomyces* sp., leads to the formation of double-length channels with predominant anion selectivity [141,142,143]. The decrease in the dipole potential (with the hydrocarbon region being positive relative to the aqueous phase) is expected to diminish the electrostatic energy at the center of the pore for cations and to increase it for anions [118]. This should cause an increase in the conductance of the cationic GrA channels and a decrease in the amplitude of the anionic SrE and AmB pores. The quantitative assessment of the decrease in the dipole potential in the presence of small molecules of plant origins (Table 1) makes it possible to calculate the changes in the conductance of the GrA, SrE, and AmB channels, taking into account the shielding of part of the dipole potential in the pores of various geometries [144]. Table 3 summarizes the mean ratios between the steady-state transmembrane currents induced by SrE, SuF, CeC, PmB, and NiS in the presence and absence of different phytochemicals. The *I_mc_/I^o^_mc_* ratio is proportional to the ratio of the steady-state number of opened single channels before and after the adsorption of the plant metabolites into the lipid bilayer.

**Figure 9:**
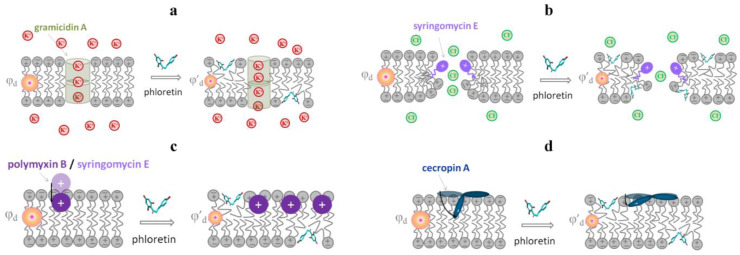
Schematic representation of the changes in the pore-forming activity of anti-microbial agents induced by decrease in the membrane dipole potential at phloretin’s addition: (**a**) an increase in the conductance of single cation-selective gramicidin A channels (colored with olive); (**b**) a decrease in the conductance of single anion-permeable syringomycin E channels (colored with violet); (**c**) facilitation of immersion of polymyxin B/syringomycin E cations (colored with purple) accompanied by an increase in the number of open channels; and (**d**) suppression of the immersion of the C-terminal domain of cecropin A (colored with dark cyan), resulting in a decrease in the number of cecropin A channels.

By analyzing the data presented in Table 2 and Table 3, one can draw the following conclusions:

(1) As expected, a reduction in the membrane dipole potential causes a decrease in the conductance of anionic channels and an increase in the conductance of cationic pores. The flavonoids phloretin and genistein, the alkaloids capsaicin and dihydrocapsaicin, and the steroid saponin tribulosin, which drastically reduce the membrane dipole potential, (Table 1) lead to an increase in the amplitude of the GrA channels, but the observed changes are small due to the significant (about 80%) shielding of the dipole potential in the aqueous pore of the GrA channel [125,144,145]. The other tested phytochemicals that are not characterized by significant dipole-modifying effects (Table 1), such as the alkaloids pentoxifylline, piperine, and synephrine and the triterpenoid sapogenin lupeol, do not practically change the conductance of the GrA channels (Table 2).
  The opposite effects are observed in the cases of the SrE and AmB channels (Table 2). The dipole-potential-diminishing polyphenols phloretin, myricetin, butein, and naringenin (Table 1) cause a significant reduction in SrE pore conductance (Table 2). The changes are not expressed due to about a 90% shielding of the membrane dipole potential in the SrE pore [145]. The high shielding of the dipole potential in the SrE pore practically eliminates the effect of the phytochemicals that reduce the dipole potential by less than 50 mV, such as 4′-hydroxychalcone, cardamonin, liquiritigenin, licochalcone A, resveratrol, pentoxifylline, piperine, and synephrine (Table 1 and Table 2). The schematic representation of the mechanism of action of phloretin in the conductance of the single GrA and SrE channels is presented in Figure 9a,b.
  The double-length AmB channels are more sensitive to the changes in the bilayer dipole potential probably due to lower shielding (about 60%) in the aqueous pore: phloretin and quercetin, which decrease the dipole potential by more than 100 mV, lead to a 2–3-fold decrease in AmB conductance (Table 1 and Table 2).
(2) In contrast to the very modest changes in the conductance of the GrA and SrE channels with the decrease in the membrane dipole potential, the changes in the lifetime of the channels are more dramatic (Table 2). Phytochemicals that diminish the membrane dipole potential might induce a several-fold increase in the dwell time of the GrA channels and a more than 100-fold reduction in the lifetime of the SrE pores. The authors of the cited publications in Table 2 attributed the changes in the lifetime of the channels to the fact that the gating particles cross the region of the potential jump during the opening/closing of the channels.
(3) A decrease in the membrane dipole potential causes a significant increase in the steady-state transmembrane currents induced by SrE, PmB, and NiS and a decrease in the pore-forming activity of SuF and CeC. Taking into account that the molecules of SrE, PmB, and NiS possess a positive net charge while SuF has a negative charge, the observed changes in the transmembrane current might be rationalized by the assumption that pore formation includes the immersion of the cations/anions of the channel-forming agents into the lipid bilayer. The decrease in the membrane dipole potential facilitates the incorporation of the cations of SrE, PmB, and NiS and inhibits the introduction of the SuF anions (Figure 9c). Despite the net positive charge of the CeC molecules, a decrease in their pore-forming ability with the diminishing membrane dipole potential might be explained by the embedment of the C-terminal domain of CeC into the lipid bilayer by its negative pole [116] (Figure 9d).

## 4. Conclusions and Outlook

In summary we made the following conclusions:

(i). Phytochemicals are able to change the membrane dipole potential through two different methods: an alteration in the membrane hydration (flavonols and saponins) and an incorporation of polar plant molecules into the membrane (chalcones/dihydrochalcones, piperine, and benzylamines).
(ii). The most significant structural features that determine the effect of phytochemicals on the membrane dipole potential include the following:
  - The glycosylation of sapogenin and flavonoid molecules;
  - The oxidation of the hydrocarbon fragment connecting the two phenolic rings in polyphenol molecules;
  - The double bond in the C-ring of flavonoids;
  - The localization of the hydrophobic substituents in xanthine molecules.
(iii). The decrease in the membrane dipole potential with a phytochemical’s addition leads to moderate changes in the conductance of single ion-selective channels and to dramatic alterations in the lifetime and number of pores formed by anti-microbial agents.

The summarized data concerning the possibility of a phytochemical influence on the transmembrane distribution of the electrical potential should be taken into account when discussing the molecular mechanisms of phytochemical’s biological and pharmacological actions, including the changes in the membrane permeability and activity of voltage-dependent integral proteins and the subsequent alterations in the signal transduction.

## Figures and Tables

**Table 1:** The quantitative characteristics of phytochemical-induced alteration in the membrane boundary and dipole potential.

Class	Phytochemical	Charge *	LogD *	µ ^$^, D	−Δφ_b_(max), mV	−Δφ_d_(max), mV	References
polyphenols ^#^	phloretin	−0.23	3.79	3.22	147 ± 7 ~220 ~190	–	[122] [100] ^@^ [124] ^Ω^
	phlorizin	−0.27	0.85	1.63	92 ± 4	nd	[122]
	4′-hydroxychalcone	−0.25	3.46	2.43	38 ± 7	35 ± 10	[117]
	butein	−0.76	2.84	5.90	120 ± 19	150 ± 12	
	cardamonin	−0.66	3.36	1.83	59 ± 12	38 ± 9	
	licochalcone A	−0.30	4.67	3.79	66 ± 12	43 ± 11	
	isoliquiritigenin	−0.75	3.15	2.69	41 ± 12	31 ± 11	unpublished data
	liquiritigenin	−0.30	2.34	0.34	66 ± 25	30 ± 13	[117]
	naringenin	−0.28	2.70	1.30	72 ± 11	73 ± 14	
	quercetin	−1.25	1.00	4.42	104 ± 7	nd	[122]
	myricetin	−1.42	0.65	4.95	111 ± 11	nd	
	rutin	−1.20	−2.02	2.51	42 ± 6	nd	
	biochanin A	−1.02	2.27	3.29	109 ± 11	nd	
	genistein	−1.06	2.12	3.81	70 ± 10	nd	
	genistin	−0.60	0.44	3.52	7 ± 2	nd	
	daidzein	−0.92	1.77	2.57	20 ± 6	nd	
	catechin	−0.03	1.78	3.67	6 ± 2	nd	
	taxifolin	−0.34	1.65	2.52	2 ± 1	nd	
	resveratrol	−0.11	3.37	0.53	11 ± 4	9 ± 5	[117]
	piceatannol	−0.12	3.06	1.59	15 ± 4	10 ± 6	unpublished data
alkaloids ^§^	caffeine	0	−0.55	3.29	2 ± 2	nd	[125]
	pentoxifylline	0	0.23	5.44	4 ± 2	nd	
	1,7–dimethylxanthine	0	0.24	3.59	23 ± 5	21 ± 6	
	3,9–dimethylxanthine	−0.02	−0.82	7.29	4 ± 3	nd	
	theophylline	−0.28	−0.89	6.53	41 ± 16	40 ± 5	
	3–isobutyl–1–methylxanthine	−0.09	0.40	6.99	22 ± 3	20 ± 9	
	7–(β–hydroxyethyl) theophylline	0	−1.24	2.26	6 ± 2	nd	
	lupinine	1.00	−1.52	1.24	3 ± 3	nd	
	cotinine	0	0.21	4.95	6 ± 2	nd	
	atropine	0.99	−0.41	3.58	4 ± 4	nd	
	quinine	0.98	0.86	2.39	26 ± 9	16 ± 6	
	berberine	1.00	−1.28	nd	3 ± 2	nd	
	piperine	0	2.78	5.37	51 ± 8	40 ± 13	
	melatonin	0	1.15	4.93	26 ± 9	15 ± 8	
	tabersonine	0.98	0.90	1.28	6 ± 2	nd	
	colchicine	0	1.46	6.53	27 ± 5	nd	
	capsaicin	0	3.75	4.66	118 ± 11	92 ± 11	
	dihydrocapsaicin	0	4.11	4.95	119 ± 12	92 ± 15	
	hordenine	0.98	0.06	0.99	29 ± 8	23 ± 11	
	synephrine	0.97	−1.39	2.32	41 ± 12	24 ± 9	
	conessine	nd	−1.45	1.81	19 ± 6	12 ± 7	
	solasodine	0.99	2.50	nd	5 ± 2	nd	
	solanidine	0.98	1.39	1.24	2 ± 2	nd	[126]
saponins and related compounds ^&^	digitonin	0	−4.96	3.79	36 ± 4	na	
	tribulosin	nd	nd	5.96	47 ± 6	nd	
	dioscin	0	1.71	4.27	39 ± 8	nd	
	diosgenin	0	4.93	1.38	6 ± 2	nd	
	escin	−1.00	−4.29	7.71	20 ± 5	nd	
	uvaol	0	6.11	1.41	1 ± 1	nd	
	lupeol	0	7.45	1.23	1 ± 1	nd	
	betulin	0	6.17	0.99	1 ± 1	nd	

*—the values of the charge and logarithm of octanol–water distribution coefficient at pH of 7.4, LogD_o/w_, were predicted by ChemAxon (Chemicalize, JChem Technology Marvin, Hungary). ^$^—the calculations of the dipole moments were performed by HyperChem 7.0 (Hypercube, Inc., Gainesville, FL, USA) by applying MNDO semi-empirical quantum chemical method with STO-3G basis. Δφ*_b_*(max)—the maximum changes in the boundary potential of the membranes at the adsorption of metabolites. The magnitudes were estimated using the ratio of K^+^-nonactin-induced membrane conductance values before and after addition of the phytochemicals as described in [118,122]. The lipid bilayers were composed of dioleoylphosphocholine (^#^), palmitoyloleoylphosphocholine (^§^), and diphytanoylphosphocholine (^&^). ^@^—this was determined using a method based on the comparison of the binding and translocation rates of hydrophobic cation [100]. ^Ω^—this was determined using a method based on the anion spin labels and the variation in the intra-membrane electric field [124]. Δφ*_d_*(max)—the maximum changes in the dipole potential of the membranes at the adsorption of metabolites were estimated using dipole-sensitive fluorescence probe, di-8-ANEPPS. nd = not determined.

**Table 2:** The effects of phytochemicals on the properties of ion channels formed by gramicidin A, syringomycin E, and amphotericin B.

Agent	Phytochemical *	Parameters		References
		*g_sc_/g^o^_sc_*	*τ_sc_/τ^o^_sc_*	
GrA	phloretin	1.4 ± 0.2	12.7 ± 4.4	[110,130]
	genistein	1.1 ± 0.1	4.3 ± 1.5	[110]
	daidzein	1.0 ± 0.1	2.4 ± 0.3	
	pentoxifylline	1.0 ± 0.1	1.2 ± 0.6	[125]
	piperine	0.9 ± 0.1	0.9 ± 0.6	
	capsaicin	1.1 ± 0.1	2.0 ± 1.3	
	dihydrocapsaicin	1.2 ± 0.1	1.6 ± 0.8	
	synephrine	1.1 ± 0.1	0.9 ± 0.6	
	tribulosin	1.1 ± 0.1	1.6 ± 0.6	[126]
	lupeol	1.0 ± 0.1	1.0 ± 0.5	
SrE	phloretin	0.6 ± 0.1	0.01 ± 0.01	[112]
	myricetin	0.6 ± 0.2	0.05 ± 0.01	unpublished data ^&^
	4′-hydroxychalcone	0.9 ± 0.2	nd	[117]
	butein	0.6 ± 0.1	nd	
	cardamonin	1.0 ± 0.2	nd	
	liquiritigenin	0.9 ± 0.2	nd	
	naringenin	0.7 ± 0.1	nd	
	licochalcone A	1.0 ± 0.2	nd	
	resveratrol	0.9 ± 0.2	nd	
	pentoxifylline	1.0 ± 0.1	1.2 ± 0.1	[125]
	piperine	1.0 ± 0.1	0.04 ± 0.01	
	capsaicin	0.9 ± 0.1	0.01 ± 0.01	
	dihydrocapsaicin	0.9 ± 0.1	0.01 ± 0.01	
	synephrine	1.0 ± 0.1	0.6 ± 0.1	
AmB	phloretin	0.3 ± 0.1	nd	[135]
	quercetin	0.6 ± 0.1	nd	

*g_sc_/g^o^_sc_* and *τ_sc_/τ^o^_sc_*—the ratio of the conductance and the ratio of the dwell time of the single channels in the presence (*g_sc_*, τ*_sc_*) and absence (*g^o^_sc_*, *τ^o^_sc_*) of phytochemicals, respectively; *—the concentration of polyphenols, alkaloids, and saponins was equal to 20, 400, and 50 μM, respectively; ^&^—membranes were composed of diphytanoyl phosphocholine and were bathed in 1.0 M NaCl at pH of 6.0; and nd = not determined.

**Table 3:** The effects of phytochemicals on the multi-channel activity of anti-microbial agents: syringomycin E, surfactin, cecropin A, polymyxin B, and nisin.

Agent	Phytochemical *	*I_mc_/I^o^_mc_*	References
SrE	phloretin	~20,000	[145]
SuF	phloretin	0.02 ÷ 0.2	[115]
CeC	phloretin	0.3 ± 0.2	[116,125]
	myricetin	1.1 ± 0.1	
	pentoxifylline	0.9 ± 0.1	
	piperine	0.3 ± 0.1	
	capsaicin	0.1 ± 0.1	
	dihydrocapsaicin	0.2 ± 0.1	
	synephrine	1.1 ± 0.4	
PmB	phloretin	28 ± 4	[133]
NiS	phloretin	5.3 ± 1.3	[134]
	capsaicin	11.3 ± 1.9	

*I_mc_/I^o^_mc_*—ratio of the transmembrane currents induced by antibiotics in the presence (*I_mc_*) and absence (*I^o^_mc_*) of phytochemicals in the bilayers at *V* = 50 mV; *—the concentration of polyphenols and alkaloids was equal to 20 and 400 μM, respectively.
